# Supplementary material for: SpliceProt 2.0: A Sequence Repository of Human, Mouse, and Rat Proteoforms
Source: Int J Mol Sci. 2024 Jan 18;25(2):1183. doi: 10.3390/ijms25021183 (PMC10816255; doi:10.3390/ijms25021183)
Supplement: Supplementary file 1 [file ijms-25-01183-s001.zip › Supplementary File S1.pdf]

Additional File 1:

TSL Filter The TSL (Transcript Support Level) transcript flag was created by Ensembl/GENCODE [55,56] to assess the annotation quality of a given transcript. In the SplicePróot 2.0 ternary matrices reconstruction step, the TSL value (TABLE S1) assigned to each transcript available in our database was used as a parameter to define whether the internal and external coordinates from first and last exons would be taken as reliable. The lower the TSL value, the greater the reliability of sequence information and splicing junctions for the transcript. In the absence of transcripts with TSL value 1 for the gene, the transcript with the lowest TSL value was chosen as the best annotated. In cases of genes with more than one transcript that have the same TSL value, the one with the longest sequence was chosen. For transcripts that did not have annotated TSL value, the value "NULL" was considered, being considered equivalent to those that had the value "NA". Thus, both "NULL" and "NA" were considered the highest values of TSL for these isolated cases. These parameters were used only for humans and mice.

Table S1: TSL Flags Classification

**TSL value**	
1	All transcript splicing junctions are supported by at least 1 reliable mRNA.
2	The transcript is supported by an unreliable mRNA or multiple ESTs.
3	The transcript is supported by a single EST.
4	The transcript is supported by an unreliable EST.
5	There is no support for the structural transcript model.
NA	The transcript was not analyzed due to any of the following reasons: it is a pseudogene annotation, a transcript from the HLA gene, a transcript from an immunoglobulin gene, a transcript from the T cell receptor, or it is a transcript with only one exon.

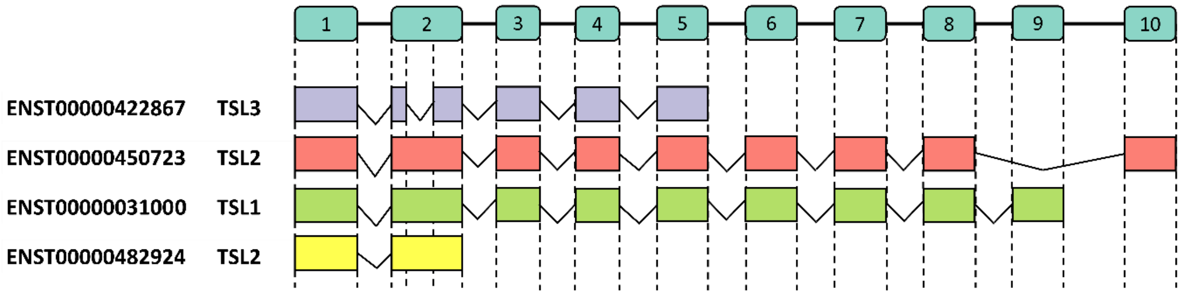

	1	2	3	4	5	6	7	8	9	10
**ENST00000422867** TSL3	|1|	|101|	|1|	|1|	|1|	|0|	|0|	|0|	|0|	|0|
**ENST00000450723** TSL2	|1|	|111|	|1|	|1|	|1|	|1|	|1|	|1||	|0|	|1|
**ENST00000031000** TSL1	|1|	|111|	|1|	|1|	|1|	|1|	|1|	|1|	|1|	|0|
**ENST00000482924** TSL2	|1|	|111|	|0|	|0|	|0|	|0|	|0|	|0|	|0|	|0|

Figure S1: REPRESENTATIVE SCHEME CONSTRUCTION OF A TERNARY MATRIX FOR GENE CYP51A1 (ENSG00000001630). The indicated gene has 4 transcripts and 3 variants. That is, ENST00000003100 and ENST00000482924 are the same variant generated by alternative splicing. The character "|" represents the limits of the exons, the character “0” represents the absence and “1” the presence of the exon regions.

APPRIS Filter The APPRIS database [32-34] was created with the GENCODE [1] consortium to perform the annotation of proteins from alternative splicing events and the transcripts that originate these proteins. For this, APPRIS has a pipeline that crosses and uses structural, functional and orthology predicted information’s (residues of highly conserved amino acids among species) available in other biological databases, such as Pfam [94] and Ensembl [28]. As with the TSL flag cited above, APPRIS also assigns values to isoforms based on predefined characteristics (TABLE S2).

In the step of reconstruction of *Rattus norvegicus* transcripts applying the methodology of ternary matrices, the APPRIS value (P1-P5 and ALT1-ALT2) was used as a parameter to define which internal and external coordinates of the exons would be classified as reliable. The transcript with the lowest APPRIS value was always selected as a reference for the reliable coordinates if the transcript did not have manual annotation performed by HAVANA [55] . Therefore, the smaller the APPRIS value, the better the annotation of splicing joins based on our method criteria. In cases where more than one transcript of the same gene had an APPRIS P1 value, both transcripts were selected. For the other values (P2-P5 and ALT1-ALT2), if more than one transcript of the same gene presented the same APPRIS value, the transcript with the longest sequence had the internal and external coordinates selected.

Table S2: Appris values and correspondent classifications

APPRIS value	
**P1**	Transcripts predicted as coders of the main functional isoform based only on the main APPRIS modules
**P2**	In cases where APPRIS is unable to choose a principal variant (about 25% of human protein-coding genes), the bank chooses one or more CDS as major variant candidates
**P3**	APPRIS is unable to choose a principal variant and there is more than one variant with different CCDS (consensus coding sequence) identifiers. The smaller the CCDS identifier, the less annotated is the transcript
**P4**	APPRIS is unable to choose a major variant and there is more than one variant with different (but consecutive) CCDS (consensus coding sequence) identifiers. In this case the largest isoform is chosen
**P5**	APPRIS is unable to choose a major variant and none of the candidate variants are annotated by CCDS. In this case APPRIS selects the largest candidate isoform as the main
**ALT1**	Candidate transcript models that are conserved in at least three species tested
**ALT2**	Candidate transcript models that APPEAR to be conserved in less than three species

Benchmarking The needle tool from EMBOSS package [37] uses the Needleman-Wunsch algorithm [95] to align two sequences based on their entire length. The best alignment is chosen based on the best possible score (sum of matches minus penalties applied in opening gaps and length of sequences). Since both SpliceProt 2.0 and OpenProt [47] use the Transeq tool from the EMBOSS package [37] to perform the *in silico* translation of the transcripts, we performed the global alignment only of the pairs of sequences corresponding to the same transcripts. Of the total sequences present in each repository, it was possible to align 78-89% and 64-40% of SpliceProt 2.0 and OpenProt 1.6 [38], respectively (tables S3 and S4). Here are the summary descriptive statistics from global alignment for human, mouse, and rat datasets from SpliceProt 2.0 and OpenProt 1.6 [38] .

Legend for histograms: A = *Rattus norvegicus* statistics; B = *Mus musculus* statistics; C = *Homo sapiens* statistics.

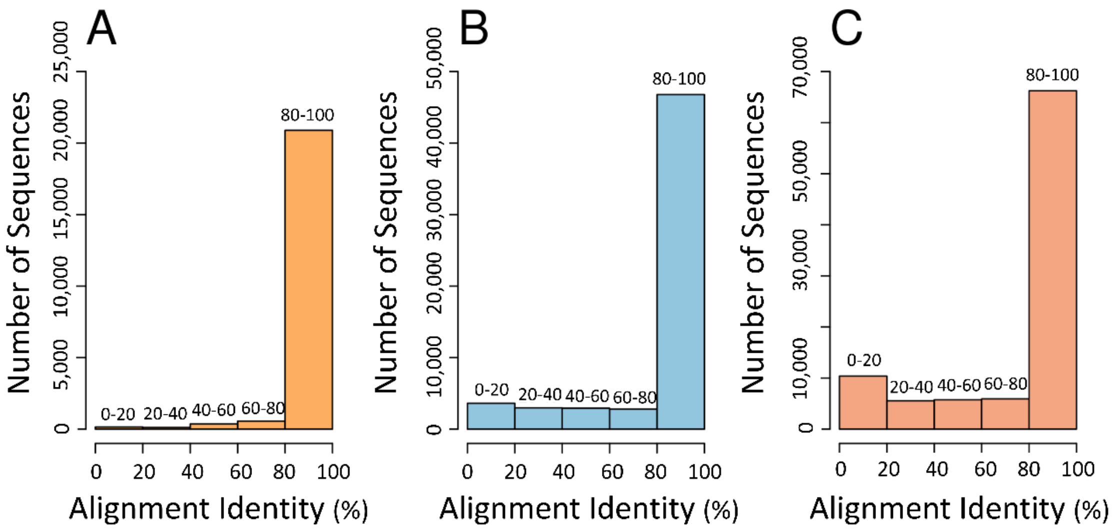

Figure S2: General alignment description. Histogram representing the distribution of identity percentages obtained in the alignment of *in silico* translations from SpliceProt 2.0 and OpenProt release 1.6. In this step, all the protein sequences available in the fasta file of both databases were aligned, if there was a correspondence between the transcripts that were translated.

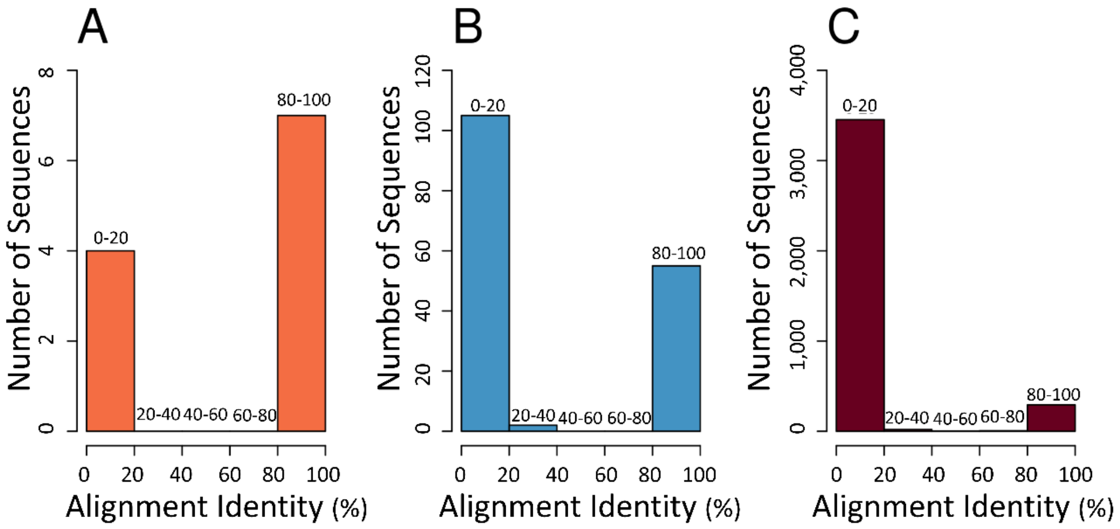

Figure S3: Alignment of proteins predicted as *New Isoforms* by OpenProt release 1.6 *versus* SpliceProt 2.0. Histogram representing the distribution of identity percentages obtained in the alignment of *in silico* translations from SpliceProt 2.0 *versus* OpenProt release 1.6.

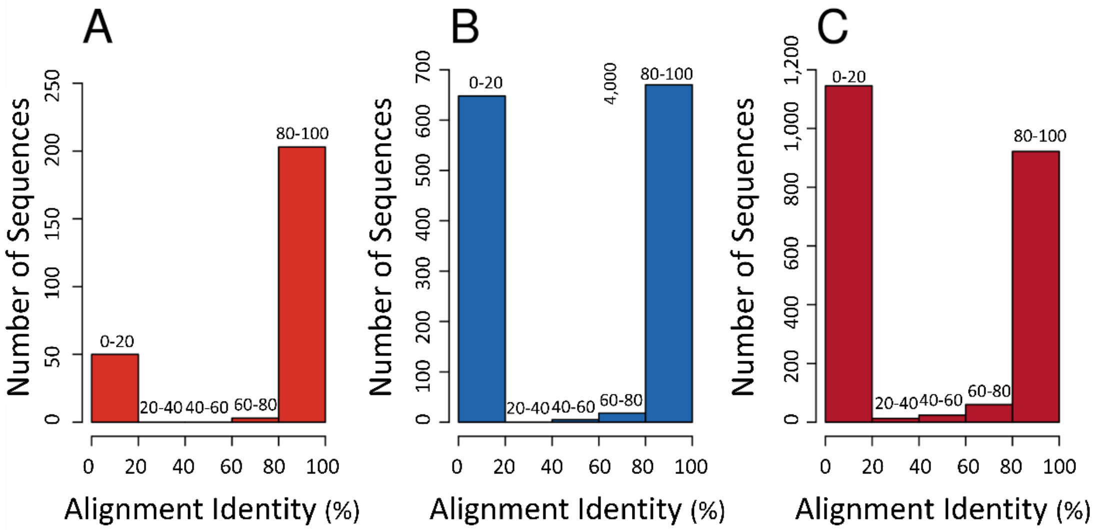

Figure S4: Alignment of proteins annotated by RefSeq and which are part of OpenProt release 1.6 *versus* SpliceProt 2.0. Histogram representing the distribution of identity percentages obtained in the alignment of *in silico* translations from SpliceProt 2.0 *versus* OpenProt release 1.6.

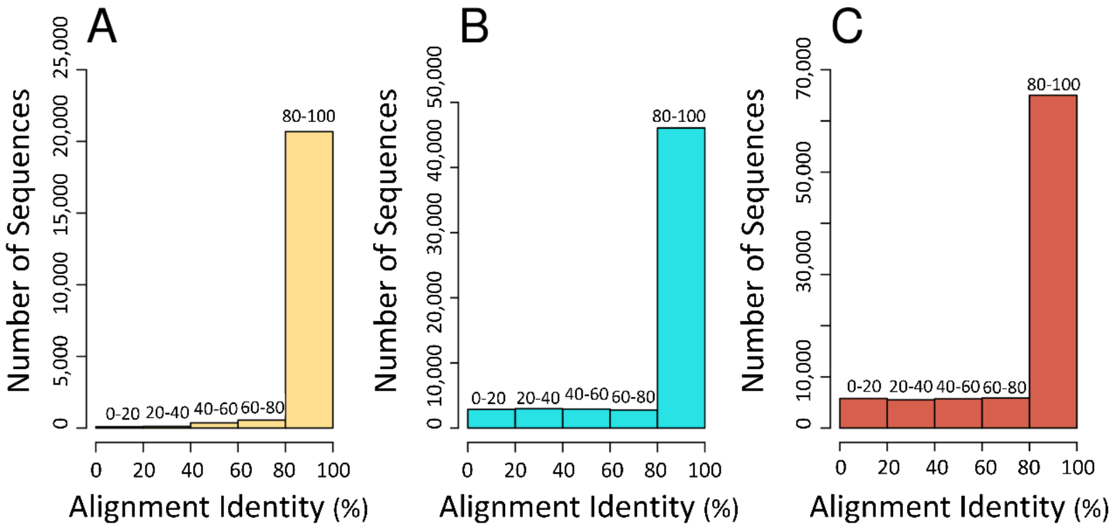

Figure S5: Alignment of reference proteins annotated by UniProtKB Consortium, and which are part of OpenProt release 1.6 versus SpliceProt 2.0. Histogram representing the distribution of identity percentages obtained in the alignment of in silico translations from SpliceProt 2.0 versus OpenProt release 1.6.

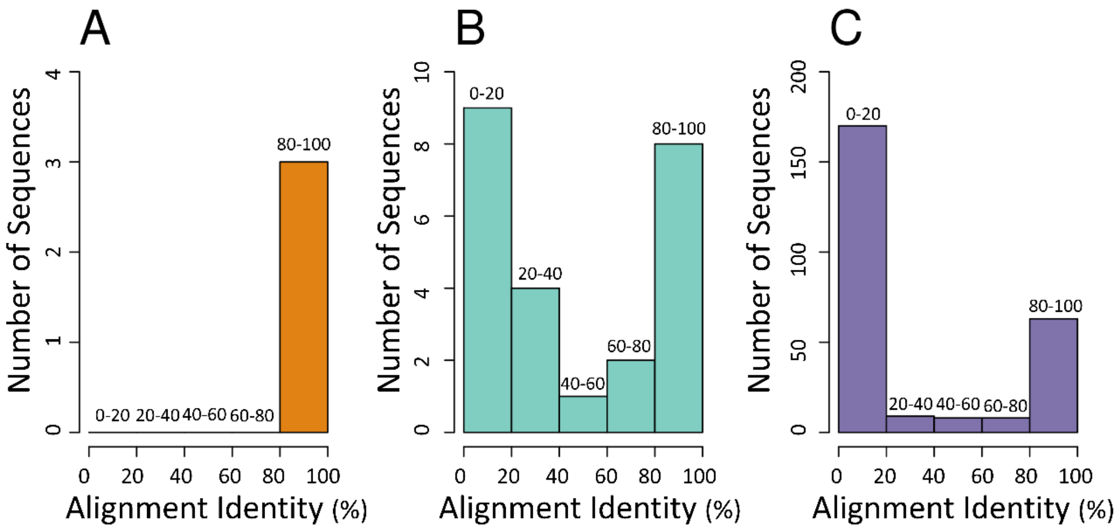

Figure S6: Alignment of proteins predicted as Alternative Proteins by OpenProt release 1.6 versus SpliceProt 2.0. Histogram representing the distribution of identity percentages obtained in the alignment of *in silico* translations from SpliceProt 2.0 versus OpenProt release 1.6.

Table S3: Number of sequences aligned and the correspondent percentage of each database (SpliceProt 2.0 and OpenProt 1.6)

	Number of shared sequences	SpliceProt 2.0	OpenProt 1.6
Human	59,090	78%	64%
Mouse	42,264	79%	63%
Rat	18,832	89%	40%

Table S4: Number of sequences aligned, and the correspondent percentage of each database aligned against SwissProt (SpliceProt 2 and OpenProt 1.6)

	SpliceProt 2.0 *versus* SwissProt		OpenProt 1.6 *versus* SwissProt	
	Number of Sequences aligned	SpliceProt 2.0	Number of Sequences aligned	OpenProt 1.6 *versus* SwissProt
Human	41,806	91%	35,811	84%
Mouse	22,651	89%	20,942	82%
Rat	4,981	51%	7,302	74%

The high standard deviation and the large variability in the distribution observed at identity levels can be explained by some peculiarities present in the analysis, mainly related to the fact that there are transcripts and proteins present in SpliceProt 2.0 that are not represented in OpenProt release 1.6 and vice versa.

Table S5: Mean and standard deviation from approximate identity values obtained in the global alignment.

**Organism**	**SpliceProt 2.0 *versus* OpenProt 1.6**		**SpliceProt 2.0 *versus* SwissProt**		**OpenProt 1.6 *versus* SwissProt**	
	Mean	SD	Mean	SD	Mean	SD
Homo sapiens	79.9	32.6	99.9	0.017	96.2	15.2
Mus musculus	87.3	26.0	99.9	0.018	98.2	9.36
Rattus norvegicus	97.0	11.0	99.9	0.009	96.2	15.2

Proteomics In order to identify the regions of the orthologous protein sequences in which the proteotypic peptides identified in the reanalysis of the shotgun proteomics datasets from healthy livers were located, an alignment was performed with the clustal omega tool [96] .

```
CLUSTAL O(1.2.4) multiple sequence alignment
Hs.168237_vn.4
MAAALQVLPRLARAPLHPLLWRGSVARLASSMALAEQARQLFESAVGAVLPGPMLHRALS   60
Mm.20258_vn.4_::_vn.2_::_vn.3_::_vn.1
MAAALQVLPCLLRAPSRPLLWGPPVARMTSGMALAEQARQLFDSAVGAVQPGPMLQRTLS   60
Rn.46307_vn.1
MAAALQVLPCLLRAPSRPFLWGPPVARMTSGMALAEQARQLFDSAVGAVQPGPMLQRTLS   60
                                            ********* * *** :*:**
***::*.***********:****** *****:*:**
Hs.168237_vn.4
LDPGGRQLKVRDRNFQLRQNLYLVGFGKAVLGMAAAAEELLGQHLVQGVISVPKGIRAAM   120
Mm.20258_vn.4_::_vn.2_::_vn.3_::_vn.1
LDPSGRQLKVRDRTFQLRENLYLVGFGKAVLGMAAAAEELLAQHLVQGVISVPKGIRAAM   120
Rn.46307_vn.1
LDPSGKQLKVRDRTFQLQENLYLVGFGKAVLGMAAAADELLGQHLVQGVISVPKGIRAAV   120

***.*:*******.***::*****************:***.****************:
Hs.168237_vn.4
ERAGKQEMLLKPHSRVQVFEGAEDNLPDRDALRAALAIQQLAEGLTADDLLLVLISGGGS   180
Mm.20258_vn.4_::_vn.2_::_vn.3_::_vn.1
EHAGKKEMLLKPHSRIQVFEGAEDNLPDRDALRAALTIQQLAEGLTADDLLLVLISGGGS   180
Rn.46307_vn.1
ELAGKQEMLLKPHSHIQVFEGAEDNLPDRDALRAAQAIQQLAERLTADDLLLVLISGGGS   180
                                            *
***:********::****************** :****** ***************
Hs.168237_vn.4
ALLPAPIPPVTLEEKQTLTRLLAARGATIQELNTIRKALSQLKGGGLAQAAYPAQVVSLI   240
Mm.20258_vn.4_::_vn.2_::_vn.3_::_vn.1
ALLPAPIPPVTLEEKQMLTKLLAARGATIQELNTIRKALSQLKGGGLAQAAYPAQVISLI   240
Rn.46307_vn.1
ALLPAPIPPVTLEEKQTLTKLLAARGATIQELNTIRKALSQLKGGGLAQAAYPAQVVSLI   240
                                            ***************
**:*********************************:***
Hs.168237_vn.4
LSDVVGDPVEVIASGPTVASSHNVQDCLHILNRYGLRAALPRSVKTVLSRADSDPHGPHT   300
Mm.20258_vn.4_::_vn.2_::_vn.3_::_vn.1
LSDVIGDPLEVIASGPTVASTHSVQDCLHILNHYGLRAALPRSVKTVLSRADSDPHGPHT   300
Rn.46307_vn.1
LSDVIGDPLEVIASGPTVASTHSVQDCLHILNHYGLRAALPRSVKTVLSRADSDPHGPHT   300

****:***:**********:*.*********:*************************
Hs.168237_vn.4
CGHVLNVIIGSNVLALAEAQRQAEALGYQAVVLSAAMQGDVKSMAQFYGLLAHVARTRLT   360
Mm.20258_vn.4_::_vn.2_::_vn.3_::_vn.1
CGHVLNVIIGSNSLALAEAQRQAEVLGYHAMVLSTAMQGDVKRVARFYGLLARVAAAHLT   360
Rn.46307_vn.1
CGHVLNVIIGSNSLALAEAQRQAEVLGYHAMVLSTAMQGDVRRVAQFYGLLARVAAACLT   360
```

****
***********.***:*:***:******: :*:******:** : **
Hs.168237_vn.4
PSMAGASVEEDAQLHELAAELQIPDLQLEEALETMAWGRGPVCLLAGGEPTVQLQGSGRG 420
Mm.20258_vn.4_::_vn.2_::_vn.3_::_vn.1
PSLAERPLEEEAELHQLAAELQLPDLQLEEALEAVVKAKGPVCLLAGGEPTVQLQGSGKG 420
Rn.46307_vn.1
SSTAERPLEEEAKLHQLAAELQLPDLQLEEALEAVAKAKGPVCLLAGGEPTVQLQGSGKG 420
* *
:**:*:**:******:**********::. .:******************:*
Hs.168237_vn.4
GRNQELALRVGAELRRWPLGPIDVLFLSGGTDGQDGPTEAAGAWVTPELASQAAAEGLDI 480
Mm.20258_vn.4_::_vn.2_::_vn.3_::_vn.1
GRNQELALHVGVELGRQPLGPIDVLFLSGGTDGQDGPTKVAGAWVMSDLISQASAESLDI 480
Rn.46307_vn.1
GRNQELALRVGAELGKQPLGPVDVLFLSGGTDGQDGPTKVAGAWVMSDLVSQASAENLDF 480
********:**.** :
****:***************:.***** :* ***:**.**:
Hs.168237_vn.4 ATFLVGHTCCT-----QG------
------------------- 493
Mm.20258_vn.4_::_vn.2_::_vn.3_::_vn.1
ATSLANNDSYTFFCRFRGGTHLLHTGLTGTNVMDVHLLILHPQ 523
Rn.46307_vn.1
ATFLDNNDSYTFFCSFQGGTHLLHTGLTGTNVMDVHLLVFHPQ 523
** * .: . *   :*

AVLGMAAAAEELLGQHLVQGVISVPK (identified exclusively in human dataset – PXD008720).
SplicePro t variant: ENSG00000168237-vn.4 [ENST00000436784]

Primary Score 5.7622

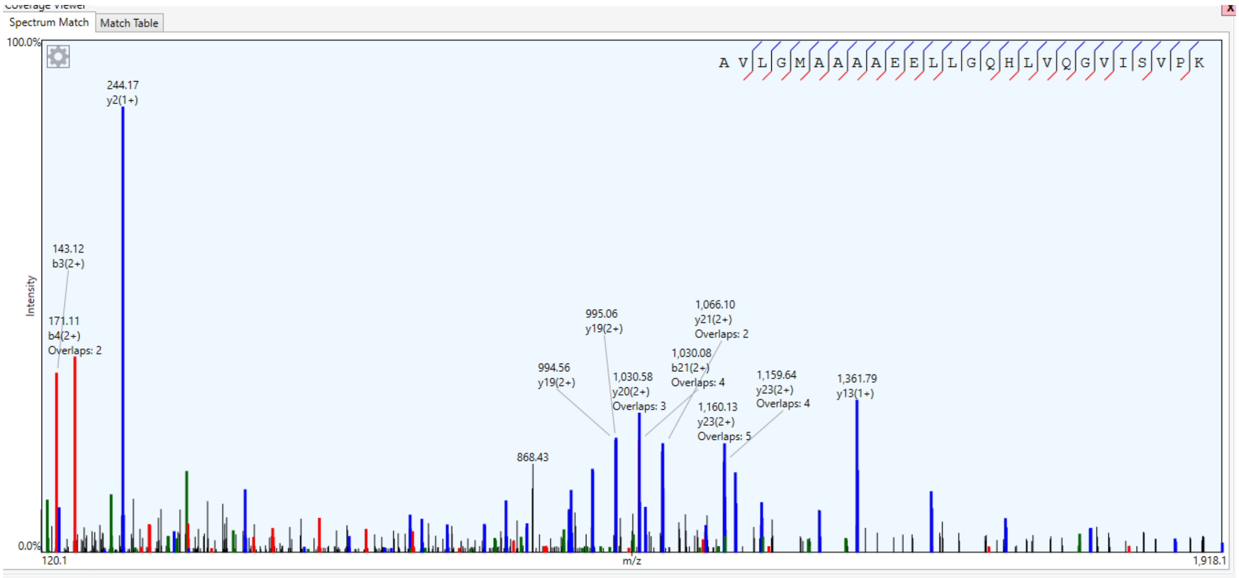

Figure S7: Spectrum match plot

LLAARGATIQELNTIRK (identified exclusively in mouse dataset – PXD020656).

SpliceProt Variants: ENSMUSG00000020258-vn.4_vn.2_vn.3_vn.1 [ENSMUST00000112543[ENSMUST00000159809[ENSMUST00000036382[ENSMUST00000162562]

Primary Score 4.7708

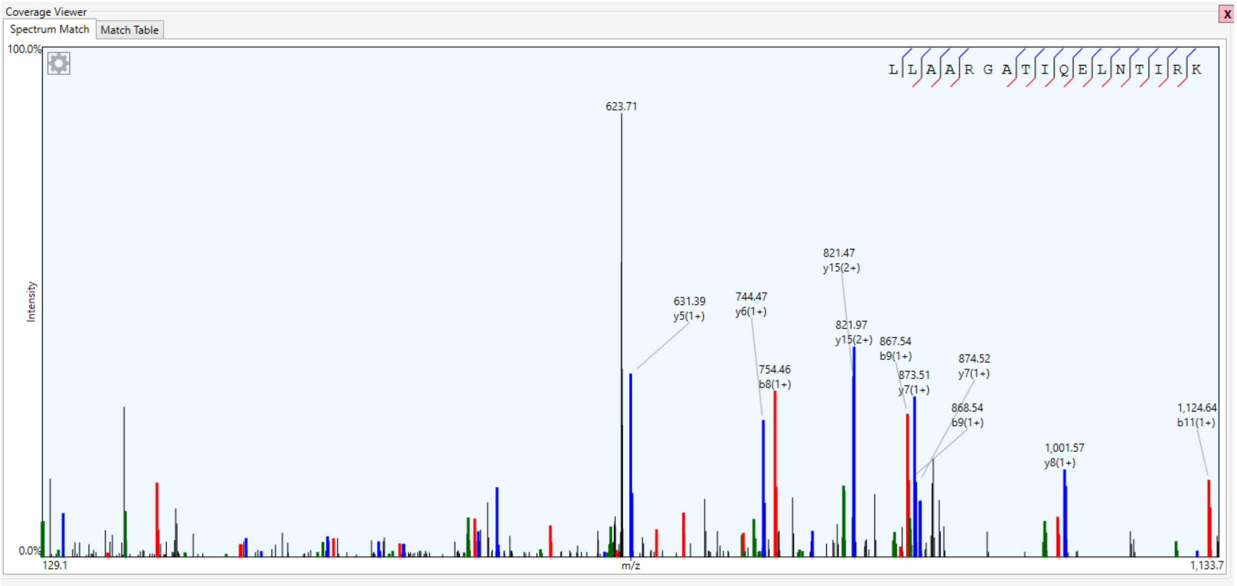

Figure S8: Spectrum match plot

LLAARGATIQELNTIRK ENSRNOG00000046307-vn.1 [ENSRNOT00000074595] **has not been identified in PLV, although the peptide appears in the sequence.**

LLAARGATIQELNTIRK ENSG00000168237-vn.4 [ENST00000436784] **has not been identified in PLV, although the peptide appears in the sequence.**

ADSDPHGPHTCGHVLNVIIGSNSLALAEAQR (identified exclusively in rat dataset – PXD016793).

SpliceProt Variant: ENSRNOG00000046307-vn.1 [ENSRNOT000000745]

Primary Score 4.8761

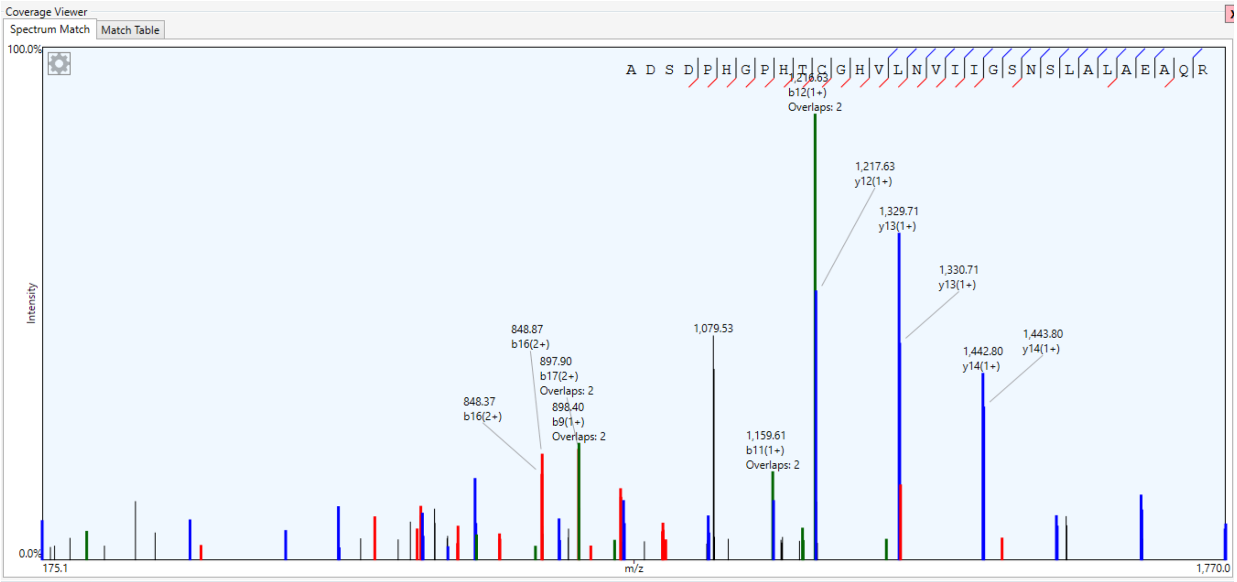

Figure S9: Spectrum match plot

ADSDPHGPHTCGHVLNVIIGSNSLALAEAQR ENSG00000168237-vn.4 [ENST00000436784] **has not been identified in PLV, although the peptide appears in the sequence.**

ADSDPHGPHTCGHVLNVIIGSNSLALAEAQR ENSMUSG00000020258vn.4_vn.2_vn.3_vn.1[ENSMUST00000112543] [ENSMUST00000159809] [ENSMUST00000036382[ENSMUST00000162562] **has not been identified in PLV, although the peptide appears in the sequence.**

GPVCLLAGGEPTVQLQGSGK identified in mouse and rat datasets (PXD020656 and PXD016793)

SplicePprot Variants (Mouse): ENSMUSG00000020258-vn.4_vn.2_vn.3_vn.1 [ENSMUST00000112543[ENSMUST00000159809[ENSMUST00000036382[ENSMUST00000162562]

Primary Score: 4.0347

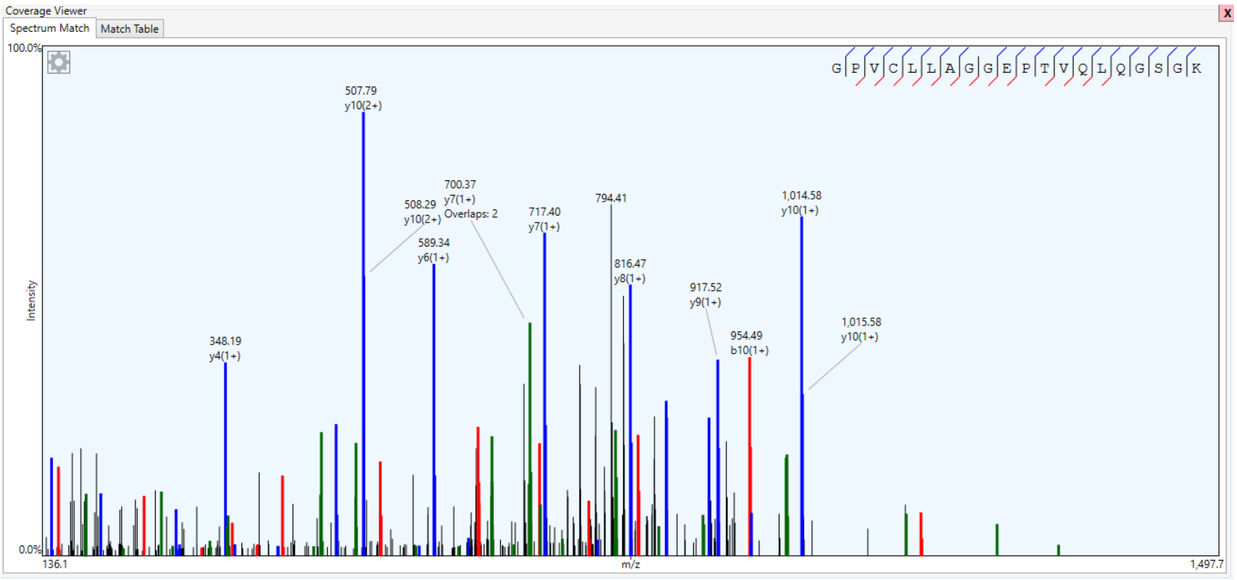

Figure S10: Spectrum match plot

SplicePprot Variants (Rat): ENSRNOG00000046307-vn.1 [ENSRNOT00000074595]

Primary Score: 4.4471

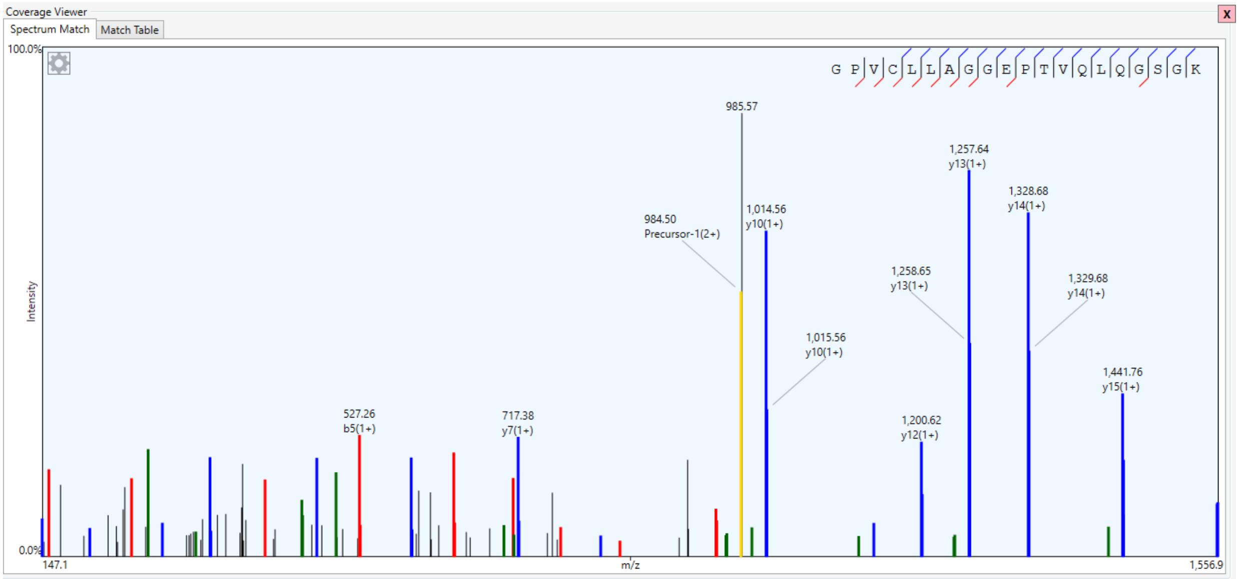

Figure S11: Spectrum match plot

******* GPVCLLAGGEPTVQLQGSGRG ********* peptide identified in human dataset PXD008720, very similar to the other, but with the substitution of a lysine for an arginine

Primary Score: 3.7241

GLYCTK

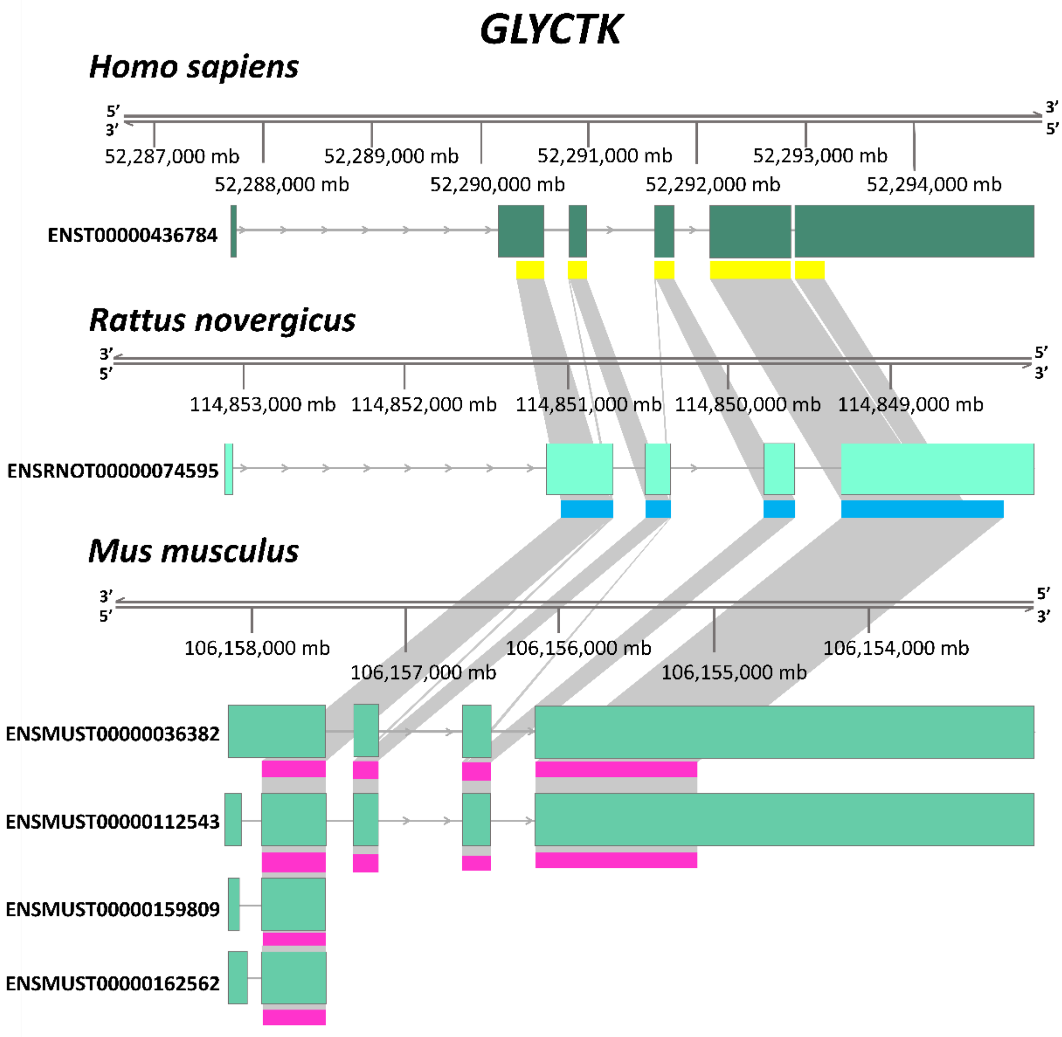

Figure S12: Graphic representation of the colocalization of the proteins from the glycerate kinase gene. Gene structures from humans, rats, and mice are represented. The representation of exon and intron structures is based on coordinates from the ternary matrices' transcript reconstruction. Exons are represented as filled boxes. We retrieved the Ensembl identifiers for each transcript to facilitate figure interpretation. The yellow, blue, and hot pink rectangles represent the protein regions with high identity scores. The gray shading illustrates the matches between orthologous protein-coding regions (exons).

NAXE and NUDT12

NAD(P)HX epimerase gene (NAXE) produces an isomerase found in all organisms catalyzing the epimerization of the S and R forms of NAD(P)HX, as well as regulating angiogenesis and accelerating the cholesterol efflux from cells to high-density lipoprotein. Nudix hydrolase 12 (NUDT12) is another enzyme that also acts in NAD+ metabolization and utilization by breaking down the pyrophosphate of nucleoside diphosphate molecules and interacting with various substrates and cofactors in different pathways [97-100] . According to the Reactome database [101] , these two enzymes – predicted here to be orthologous between humans, mice, and rats – participate in the nicotinate and nicotinamide metabolism pathway [102,103] . Regarding the structure of the genes that give rise to these proteins in humans, mice, and rats, NAD(P)HX Epimerase and nudix hydrolase 12 are transcribed with a similar structure due to the number of exons and equivalence between the coding regions (FIGURES S13–14 – Supplementary Materials).

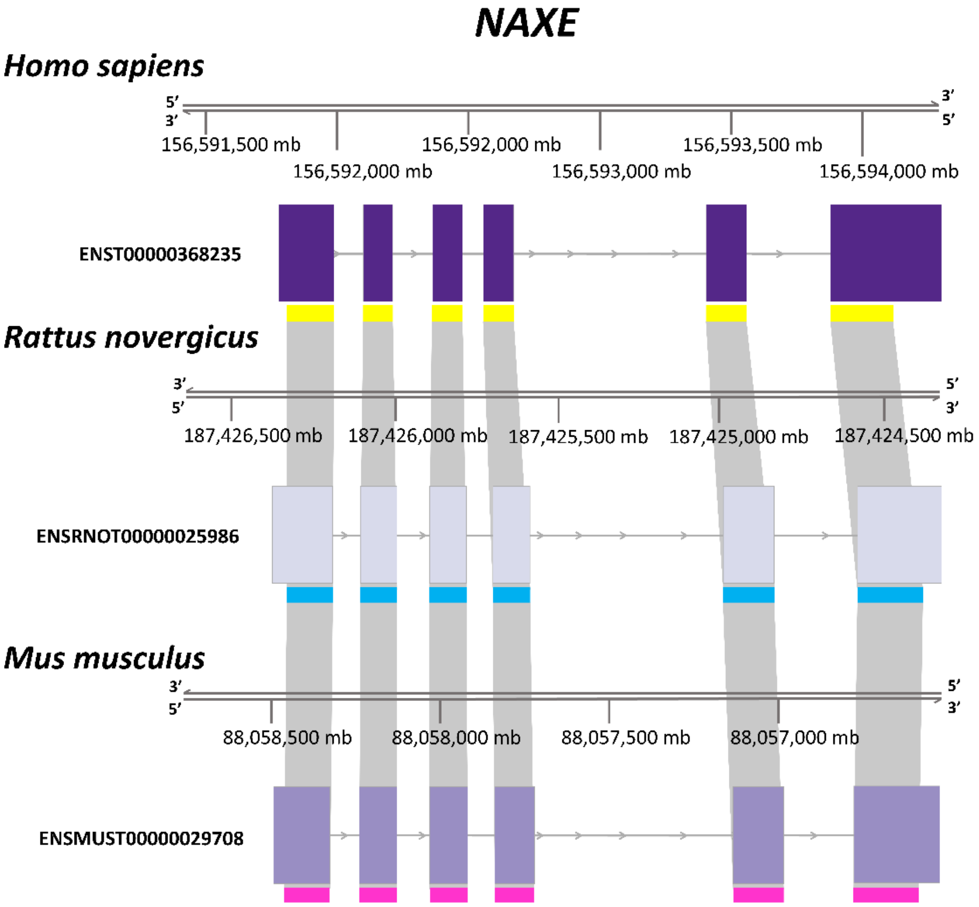

Figure S13: Graphic representation of the colocalization of the proteins from the NAD(P)HX epimerase gene. Gene structures from humans, rats, and mice are represented. The representation of exon and intron structures is based on coordinates from the ternary matrices' transcript reconstruction. Exons are represented as filled boxes. We retrieved the Ensembl identifiers for each transcript to facilitate figure interpretation. The yellow, blue, and hot pink rectangles

represent the protein regions with high identity scores. The gray shading illustrates the matches between orthologous protein-coding regions (exons).

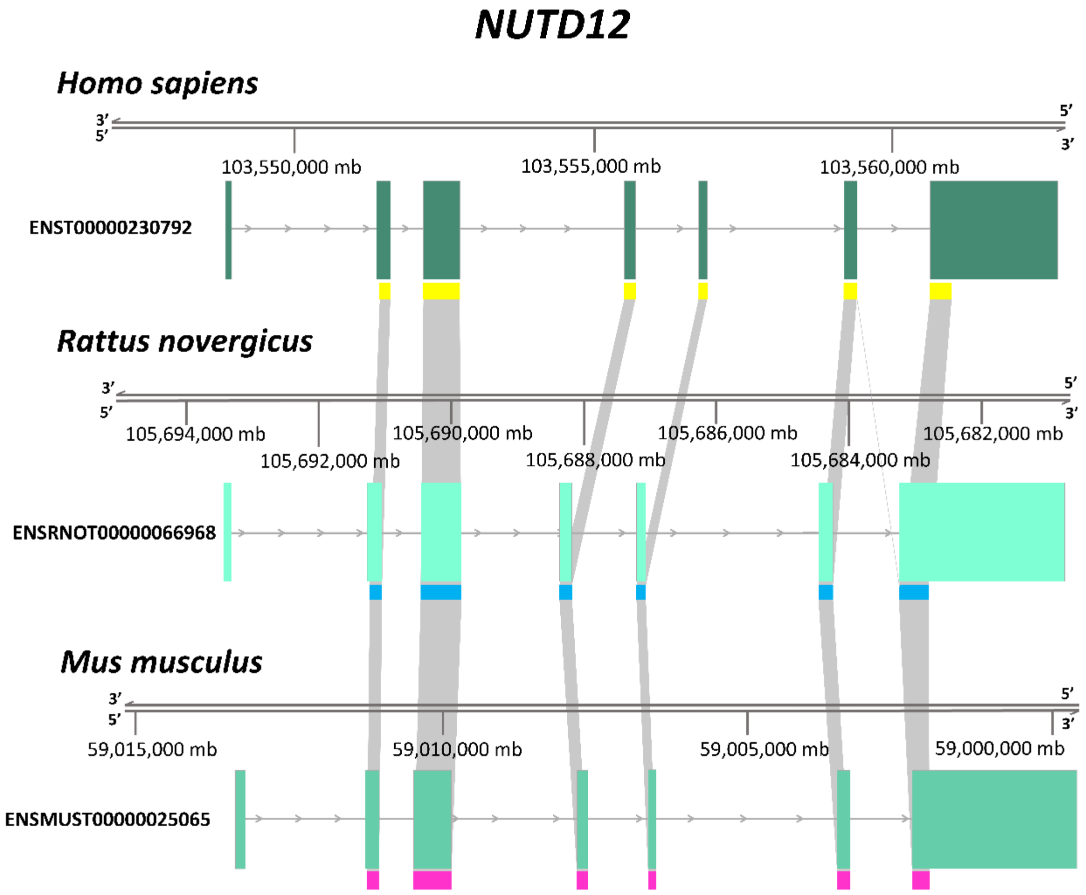

Figure S14: Graphic representation of the colocalization of the proteins from nudix hydrolase 12. Gene structures from humans, rats, and mice are represented. The representation of exon and intron structures is based on coordinates from the Ternary Matrices transcript reconstruction. Exons are represented as filled boxes. We retrieved the Ensembl identifiers for each transcript to facilitate figure interpretation. The yellow, blue, and hot pink rectangles represent the protein regions with high identity scores. The gray shading illustrates the matches between orthologous protein-coding regions (exons).

YWHAB

The protein encoded by the YWHAB gene is part of the 14-3-3 protein group composed of acidic biomolecules that are highly conserved in different species, and which show multiple functions in different tissues, like the brain, retina, and liver, in healthy humans and rats [104-111] . According to the Kyoto Encyclopedia of Genes and Genomes [106], all the 14-3-3 proteins identified here as being orthologous between humans, mice, and rats participate in the same pathway associated with basic cellular mechanisms (e.g., meiosis and MAPK signaling pathway) or diseases (e.g., hepatitis B, hepatitis C, and viral carcinogenesis). The structure of YWHAB gene that encodes the 14-3-3 proteins predicted as orthologous in this study (FIGURE S15), we found a high level of similarity in gene structure. Other interesting features include the encoding regions of the transcripts, whose hypothetical translation code to these proteins is also very similar, and the fact that our methodology pointed out the human and orthologous mouse proteins as produced by two different transcripts.

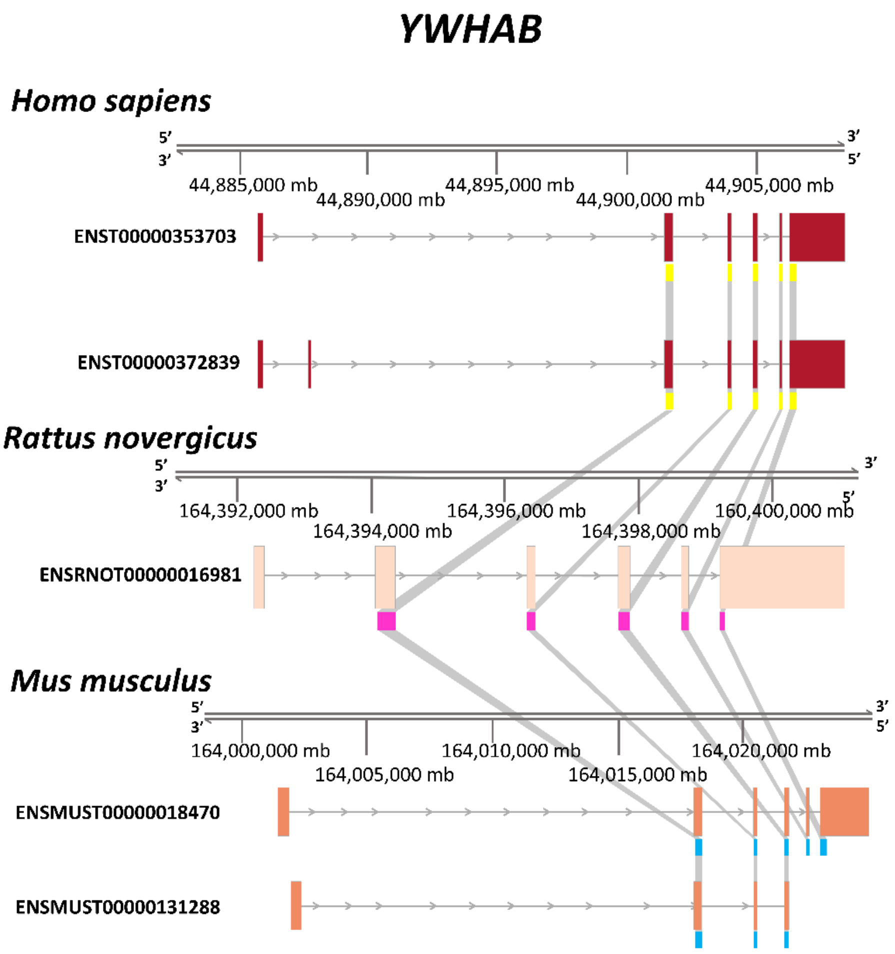

Figure S15: Representation of the colocalization of the proteins from tyrosine 3-monooxygenase/tryptophan 5-monooxygenase activation protein beta gene. Gene structures from humans, rats, and mice are represented. The representation of exon and intron structures is based on coordinates from the ternary matrices transcript reconstruction. Exons are represented as filled boxes. We retrieved the Ensembl identifiers for each transcript to facilitate figure

interpretation. The yellow, blue and hot pink rectangles represent the protein regions with high identity scores. The gray shading illustrates the matches between orthologous protein-coding regions (exons).

PSMC2

Another example of a protein generated by more than one transcript of the same gene shown to be orthologous between humans, mice, and rats is the proteasome 26S subunit, ATPase 2 (PSMC2). This is an ATPase associated with several cell cycle-related processes, such as removing misfolded proteins or those no longer helpful for cellular homeostasis [106-108] . The structure of the transcripts reconstructed by the ternary matrix methodology that originated the PSMC2, pointed out as orthologous in this study, are very similar if we observe the ordering and number of exons (Figure S16 – Supplementary Materials).

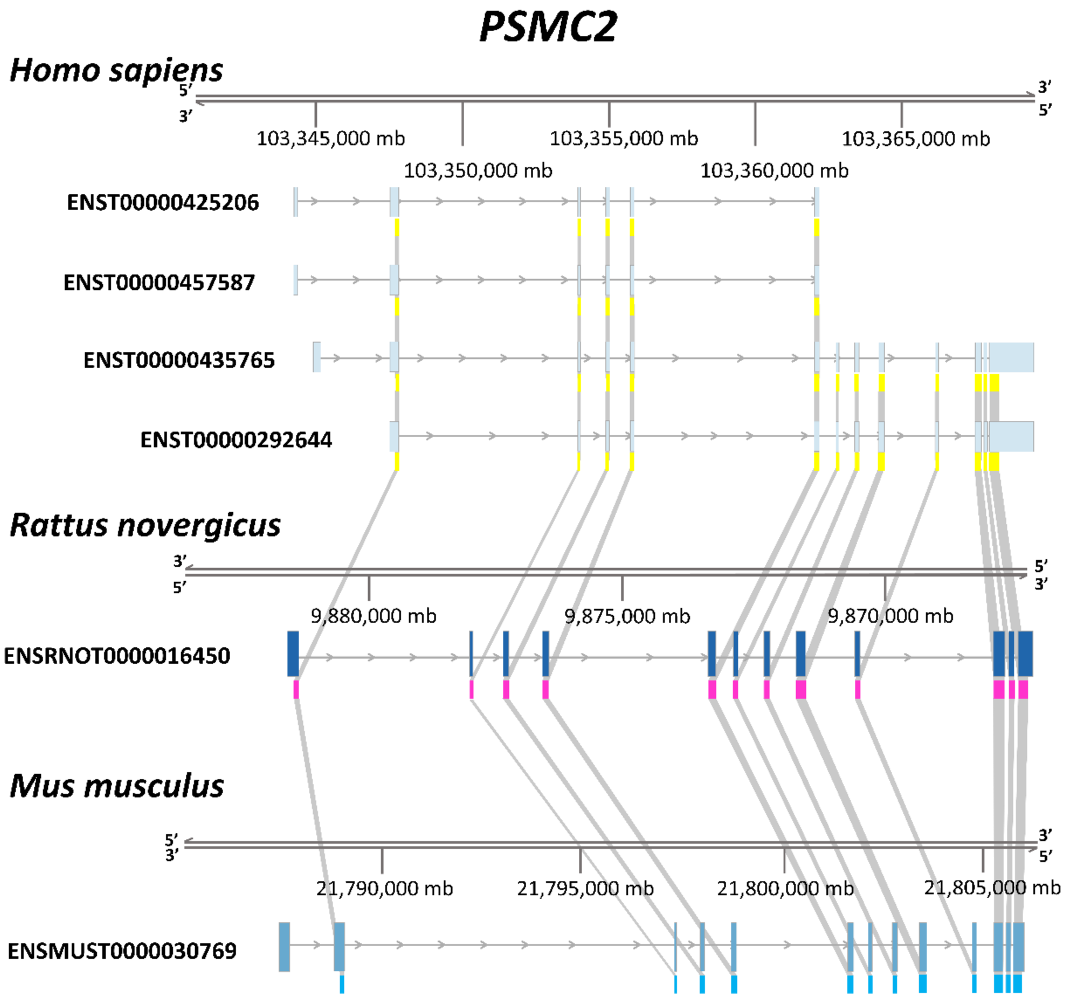

Figure S16: Graphic representation of the colocalization of the proteins from proteasome 26S subunit gene. Gene structures from humans, rats, and mice are represented. The representation of exon and intron structures is based on coordinates from the Ternary Matrices transcript reconstruction. Exons are represented as filled boxes. We retrieved the Ensembl identifiers for each transcript to facilitate figure interpretation. The yellow, blue, and hot pink rectangles represent the protein regions with high identity scores. The gray shading illustrates the matches between orthologous protein-coding regions (exons).
